# piggyPrime: High-Efficacy Prime Editing in Human Cells Using piggyBac-Based DNA Transposition

**DOI:** 10.3389/fgeed.2021.786893

**Published:** 2021-11-12

**Authors:** Jonas Holst Wolff, Jakob Haldrup, Emil Aagaard Thomsen, Sofie Andersen, Jacob Giehm Mikkelsen

**Affiliations:** Department of Biomedicine, Aarhus University, Aarhus, Denmark

**Keywords:** prime editing, CRISPR, prime editing guide RNA, pegRNA, piggyBac, DNA transposition

## Abstract

Prime editing is a novel genome editing technology that allows a wide range of tailored genomic alterations. Prime editing does not involve homologous recombination, but suffers from low efficacy. Here, we demonstrate piggyPrime, a transfected single-vector system based on piggyBac DNA transposition for genomic integration of all prime editing components in human cells allowing easy and effective transgenesis with prime editing efficacies up to 100% in cell lines.

## Introduction

Prime editing, based on the CRISPR-Cas9 system (Jinek et al., 2012; Jinek et al., 2013; Ran et al., 2013), enables precise editing of the genome by supporting targeted insertions, deletions, or any of the 12 possible single-base substitutions. Gene editing by prime editing involves neither donor templates nor double-stranded breaks (Anzalone et al., 2019). These unique properties of prime editing are based on the delivery of a prime editor (PE), consisting of a Cas9-reverse transcriptase fusion protein (hereafter referred to as PE2), along with the prime editing guide RNA (pegRNA) that specifies both the genomic target as well as the desired edit to be written directly into the genome. Prime editing has tremendous potential for treatment of disease-causing mutations, as well as generation of disease models, both *in vitro* and *in vivo* (Schene et al., 2020; Jang et al., 2021; Kim et al., 2021; Liu et al., 2021; Park et al., 2021; Petri et al., 2021; Qian et al., 2021). However, the use of prime editing is currently challenged by low efficacy, leading to time-consuming optimization and/or screening approaches in order to achieve satisfactory editing activities (Liu et al., 2020; Schene et al., 2020; Chemello et al., 2021; Kim et al., 2021; Petri et al., 2021).

Stable integration of gene cassettes encoding conventional CRISPR effectors, like Cas9 and single guide RNAs, into the genome of mammalian cells is widely used across life science research, including for generation of model cell lines and in CRISPR screens (Shalem et al., 2014; Holmgaard et al., 2017; Thomsen et al., 2020). For prime editing, effective integration of the PE2-expressing cassette into the genome of mammalian cells is challenged by the large size of the PE2 coding sequence (6351 bp). This makes the use of viral vectors difficult due to restricted packaging capabilities (Kumar et al., 2001), and so far, the PE2 system has only been integrated into the genome of mammalian cells by delivering intein-split PE2 cassettes using two separate lentiviral vectors (Anzalone et al., 2019). Here, we present piggyPrime, a non-viral, single-vector system for easy and efficient integration of all prime editing components in human cells, utilizing the large integration capacity of the piggyBac transposon system. Importantly, prolonged expression of PE2 and pegRNA facilitated by DNA transposition supports increased levels of prime editing, providing thus a novel approach for effective transgenesis.

## Materials and Methods

### Plasmid Constructions

DNA amplification was performed using Phusion High-Fidelity PCR Master Mix (ThermoFisher Scientific) unless otherwise stated. All oligoes for pegRNAs and nicking sgRNAs were from Integrated DNA Technologies. Cloning oligoes for previously published pegRNAs were derived using pegIT (Anderson et al., 2021), which was also used to design the ngRNA used for the HBB(E7V) target. pCMV-PE2 (Addgene plasmid no. 132775) and pU6-pegRNA-HEK3-CTTins (Addgene plasmid no. 132778) were gifts from David Liu (Anzalone et al., 2019). pCMV-hyPBase is described elsewhere (Yusa et al., 2011). For generation of pPBT-PE2-PGK-Blast (Addgene plasmid no. 173219), CMV-PE2 was first amplified from pCMV-PE2 using Platinum SuperFi II High-Fidelity DNA Polymerase (ThermoFisher Scientific) and assembled into a HindII-/NdeI-digested pPBT-EFS-Cas9-P2A-mCherry (unpublished) using NEBuilder® HiFi DNA Assembly Master Mix (New England Biolabs) to generate pPBT-PE2. Fragments containing PGK-Blast and a bGH poly A signal were then amplified from pCW-Cas9-Blast (Addgene plasmid no. 83481) and pCMV-PE2, respectively, using PCR and assembled into XbaI-digested pPBT-PE2. For generation of pPBT-pegRNA-Puro, a modified pegRNA Golden Gate cloning cassette was amplified from pU6-pegRNA-GG-acceptor (Addgene plasmid no. 132777), with primers designed to convert the BsaI restriction sites to BsmBI sites, which allows pegRNAs to be cloned as described elsewhere (Anzalone et al., 2019), but with the use of BsmBI (New England Biolabs). The EF-1*α* promoter was then amplified from lentiGuide-Puro (Addgene plasmid no. 52963) and assembled with the pegRNA cloning cassette into a HindIII-/SmaI-digested pPBT-EFS-Cas9-P2A-mCherry backbone using NEBuilder. The resulting plasmid, pPBT-peRNA_GG-Puro (Addgene plasmid no. 173220), was then subjected to pegRNA Golden Gate cloning of the HEK3-CTTins pegRNA using the protocol described elsewhere (Anzalone et al., 2019), but with the use of BsmBI-v2 (New England Biolabs) (Supplementary Note S2). For generation of piggyPrime vectors, pPBT-PE2-PuroTK-pegRNA-GG (Addgene plasmid no. 173222) was first generated, into which pegRNAs can easily be cloned analogous to cloning of pegRNAs into pPBT-pegRNA_GG-Puro. For this, the M-MLV RT was amplified from pCMV-PE2, P2A-PuroTK-pA was amplified from pPBT-EFS-Cas9-P2A-PuroTK (unpublished) and the modified pegRNA Golden Gate cassette was amplified from pPBT-pegRNA_GG-Puro. pPBT-EFS-Cas9-P2A-PuroTK contains a mutation within the PuroTK gene that removes a BsmBI restriction site. The three fragments were then assembled into a BamHI-/SmaI-digested pPBT-PE2-PGK-Blast using NEBuilder. All piggyPrime vectors were subsequently generated by Golden Gate assembly of pegRNAs using BsmBI-v2 (Supplementary Note S2). For generation of multiplexed piggyPrime vectors, the HBB(E7V)-piggyPrime vector was linearized using XbaI (ThermoFisher Scientific). The HEK3-CTTins pegRNA expression cassette was then amplified from the HEK3-CTTins piggyPrime vector and inserted into the HBB(E7V)-piggyPrime using NEBuilder. Same procedure was used to insert the HBB(E7V) nicking sgRNA instead, which was amplified from a pU6-HBB(E7V)-ngRNA plasmid. The primers used for cloning of all plasmids are listed in Supplementary Table S3.

### Cell Culture Conditions and Transfection

**FIGURE 1:**
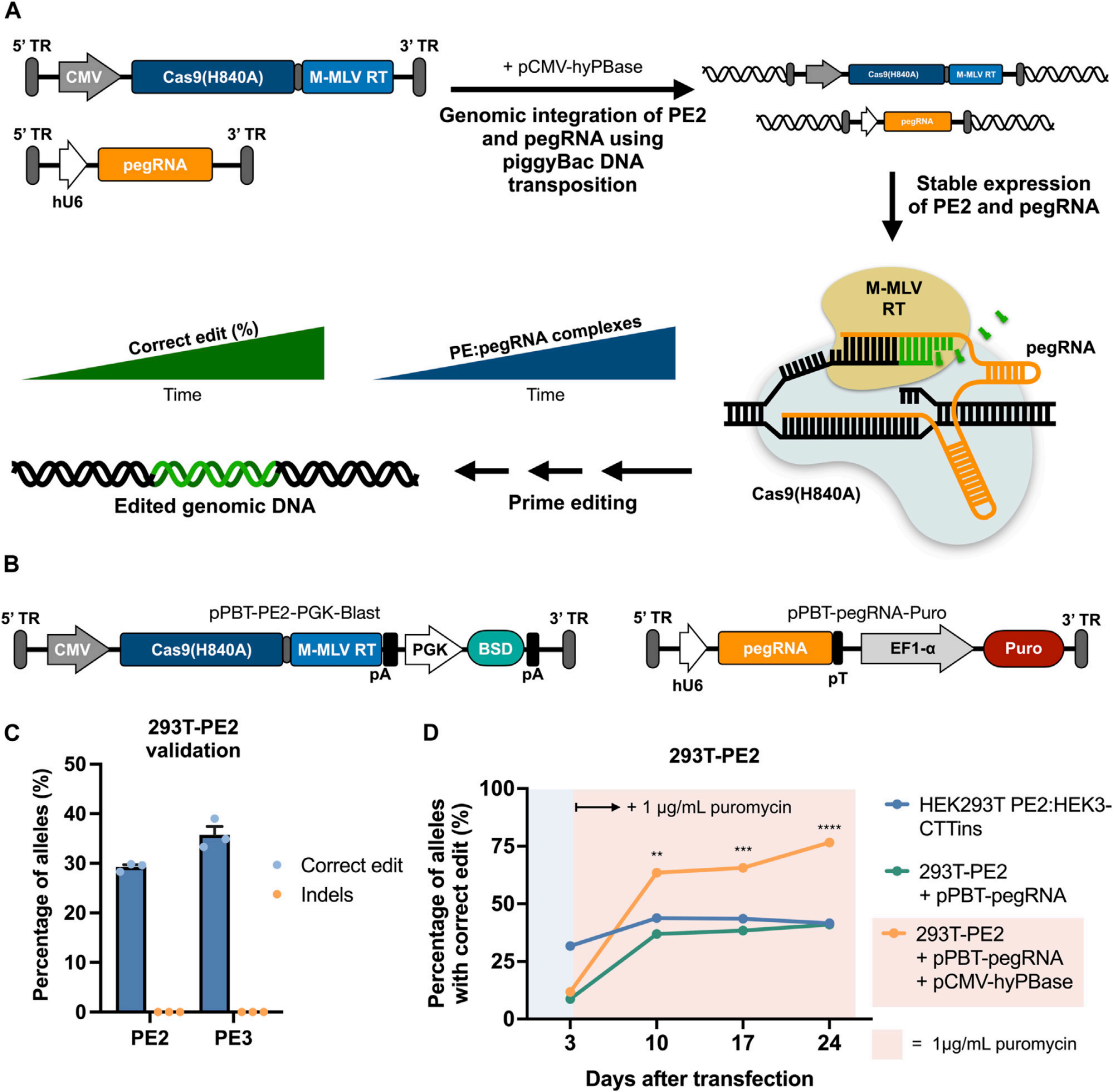
| Prime editing by piggyBac-mediated transposition of PE2 and pegRNA cassettes using separate piggyBac vectors. **(A)** Graphical overview of prime editing based on piggyBac DNA transposition, demonstrating the concept of potent editing as a result of stable expression of both PE2 and pegRNA. **(B)** Schematic representation of the piggyBac vectors carrying either PE2 (left) or a pegRNA (right). **(C)** Integration of the Cas9-RT expression cassette alone into HEK293T cells facilitates correct editing at the HEK3 target when transfected with a HEK3-CTTins pegRNA (PE2) as well as when transfected with an additional nicking sgRNA (PE3). Editing rates were determined 3 days after transfection. **(D)** Integration of the HEK3-CTTins pegRNA expression cassette into 293T-PE2 cells (293T-PE2 + pPBT-pegRNA + pCMV-hyPBase) results in increased prime editing over time compared to cells with transiently expressed PE2 and pegRNA (HEK293T PE2:HEK3-CTTins) or transiently expressed pPBT-pegRNA-Puro (293T-PE2 + pPBT-pegRNA). Puromycin was applied at day 3 after transfection only to cells with stably integrated pegRNA cassette. Data and error bars show mean (*n* = 3) ± sd. Statistical significance was calculated using multiple unpaired *t*-tests with correction for multiple testing (**p < 0.002, ***p < 0.0002, ****p < 0.0001).

HEK293T and HeLa cells (ATCC) were maintained in Dulbecco’s modified Eagle’s medium (DMEM) supplemented with 5% Fetal Calf Serum (FCS) and 1% penicillin/streptomycin (P/S). K562 cells (ATCC) were maintained in RPMI-1640 medium (Sigma-Aldrich) supplemented with 10% FCS and 1% P/S. All cells were incubated, maintained, and cultured at 37°C with 5% CO_2_. For transfection of HEK293T and HeLa, 5 × 10^4^ cells were seeded in 24-well plates 18–24 h prior to transfection. Transfection was performed using 1,000 ng of plasmid DNA and 2.5 μL TurboFect^TM^ Transfection Reagent (ThermoFisher Scientific) following the manufacturer’s instructions. For transfection of K562 cells, 1 × 10^5^ cells were seeded in 24-well plates and transfected with 1,000 ng plasmid DNA using 3 μL Lipofectamine^TM^ 2000 (ThermoFisher Scientific) according to manufacturer’s instructions. For co-transfections using the PE3 system (Figure 1C), a plasmid ratio of 3:1 (pegRNA:ngRNA) was used. For co-transfections of piggyBac vectors and pCMV-hyPBase, a plasmid ratio of 9:1 was used (vector:hyPBase). In all transfection experiments, medium was changed 16 h after transfection, and unless otherwise specified, cells were harvested 72 h after transfection. Selection medium (5 μg/ml blasticidin and/or 1 μg/ml puromycin (ThermoFisher Scientific)) was applied to indicated experiments at day 3 after transfection and maintained for the full duration of all experiments. Cells were passaged as required.

### Genomic DNA Extraction and Analysis of Prime Editing Events

Genomic DNA was extracted by addition of 100–300 μL lysis buffer (10 mM Tris-HCl, pH 7.5; 0.05% SDS; 25 μg/ml proteinase K (ThermoFisher Scientific)) depending on confluency of cells at time of harvest. Lysis mixture was incubated 2 h at 37°C followed by enzyme inactivation at 80°C for 30 min. PCR was then performed on 150 ng of extracted genomic DNA using Phusion High-Fidelity PCR Master Mix (ThermoFisher Scientific) with primers listed in Supplementary Table S2. PCR amplicons were purified by 1% agarose gel electrophoresis using E.Z.N.A® Gel Extraction Kits (Omega Bio-Tek). Sanger sequencing of PCR amplicons was then performed by Eurofins Genomics, and prime editing events were analyzed using DECODR (Bloh et al., 2021).

### Copy-Number Determination Using ddPCR

For copy-number (CN) determination of integrated piggyPrime cassettes, genomic DNA was harvested by ethanol precipitation followed by HindIII (ThermoFisher Scientific) digestion for 1 h at 37°C. Quantitative Droplet Digital PCR (ddPCR) was then performed on a QX200TM Droplet DigitalTM PCR System with ddPCR Supermix for Probes (No dUTP) (BioRad) using 25 ng of digested genomic DNA as input. Primers and probes used are listed in Supplementary Table S4 and targets the Puromycin and albumin (ALB) genes. Data were analyzed using QuantaSoft^TM^ Analysis Pro.

### pegRNA Expression-Levels ddPCR

Total RNA was extracted from cells using Roche High Puro miRNA Isolation Kit (Roche Applied Science) and subjected to DNase I treatment (ThermoFisher Scientific). 250 ng RNA was then used for cDNA synthesis using Maxima H Minus cDNA Synthesis Master Mix (ThermoFisher Scientific). Quantitative Droplet Digital PCR (ddPCR) was then performed on a QX200TM Droplet DigitalTM PCR System with ddPCR Supermix for Probes (No dUTP) (BioRad) with 1/8 diluted DNase I treated RNA as input. Primers and probes used are listed in Supplementary Table S4.

### Statistical Analysis

For all graphs, mean (*n* = 3) and standard deviation (sd) were calculated and plotted using GraphPad Prism 9. Statistical analysis was performed using multiple unpaired *t*-tests with correction for multiple testing.

## Results

### Stable Genomic Integration of Prime Editing Effectors Using piggyBac DNA Transposition Enables Increased Editing in HEK293T Cells

We first sought to establish evidence of functional integration of both PE2- and pegRNA-expressing cassettes with the use of piggyBac DNA transposition and validate that expression of the prime editing components from integrated cassettes was sufficient to confer targeted prime editing (Figure 1A). To do this, we constructed a piggyBac vector (pPBT-PE2-PGK-Blast) carrying a PE2 cassette driven by a CMV promoter as well as a blasticidin resistance gene driven by a PGK promoter (Figure 1B). We then transfected HEK293T cells with this vector along with plasmid DNA encoding a hyperactive piggyBac transposase (Yusa et al., 2011) (hyPBase), selected for blasticidin-resistant cells, and validated functional prime editing in these cells (referred to as 293T-PE2) after subsequent transfection with a plasmid encoding a pegRNA designed to incorporate a three-nucleotide CTT insertion at the *HEK3* genomic site (Anzalone et al., 2019) (HEK3-CTTins pegRNA) (Figure 1C). This resulted in editing rates that were comparable with previous reported editing efficacies using the same pegRNA (Anzalone et al., 2019). We then constructed a piggyBac vector containing the HEK3-CTTins pegRNA expression cassette (pPBT-pegRNA-Puro) (Figure 1B), integrated this into 293T-PE2 cells using hyPBase and measured the edit rates at fixed timepoints following transfection and in the presence of puromycin (Figure 1D). At day 10 after transfection, the cells with both PE2 and pegRNA cassettes integrated into the genome showed a markedly higher editing rate compared to wild-type HEK29T cells co-transfected with pCMV-PE2 and pU6-pegRNA (63.5 ± 1.4% vs 43.8 ± 2.3%) (Figure 1D). From day 10 until day 24 after transfection, the cells with integrated PE2 and pegRNA cassettes showed an increase in correct editing, whereas transiently transfected cells did not show an increase in editing, indicating that long-term expression allowed for targeted edits to accumulate. Furthermore, we did not detect any indel formation, even 24 days after transfection (Supplementary Figure S1).

### Integration of all Prime Editing Components Using All-In-One Single Vectors Allows for Potent Editing at Target Sites

**FIGURE 2:**
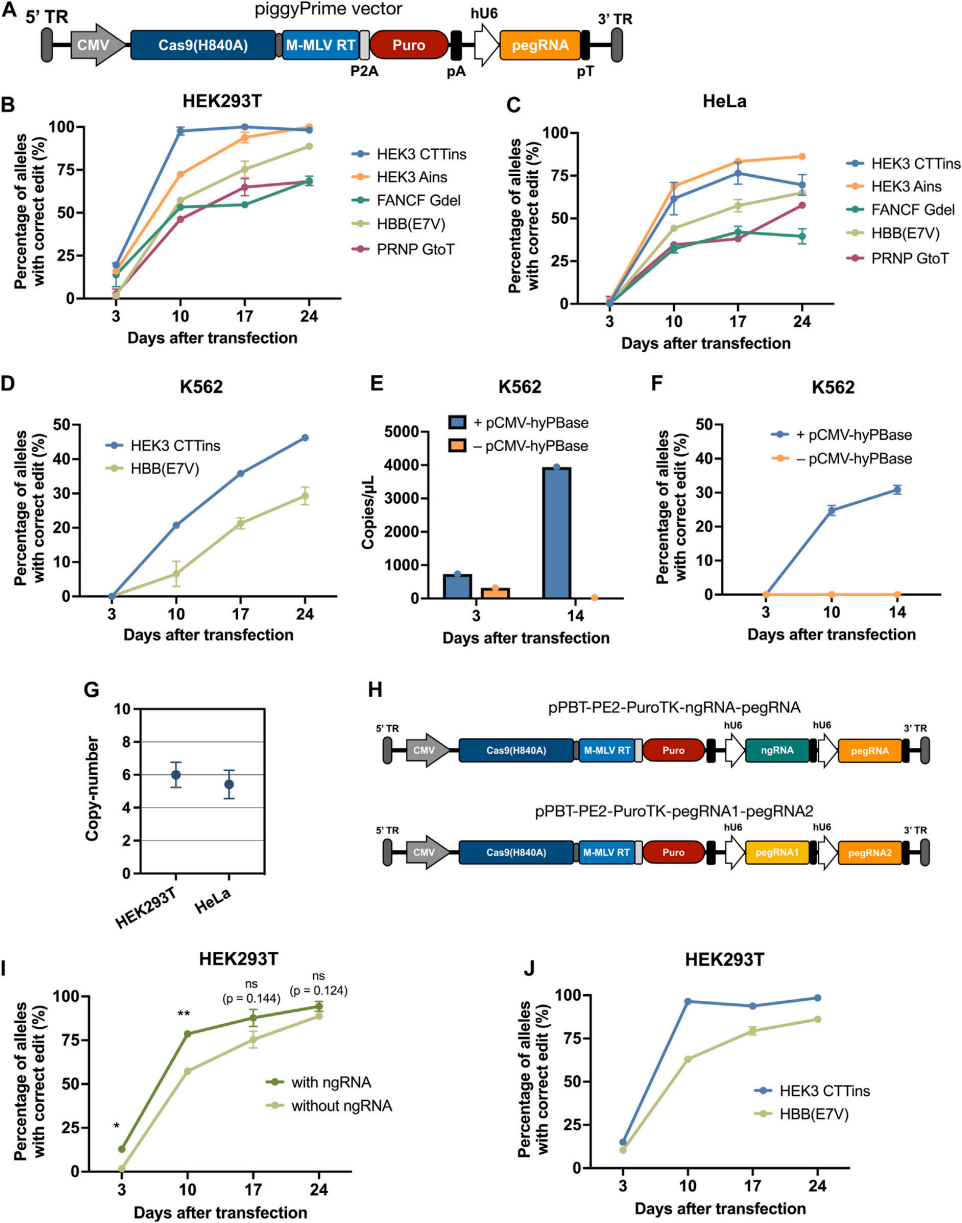
| Effective prime editing by piggyBac-mediated integration of all prime editing components using a single-vector system. **(A)** Schematic overview of the piggyPrime vector, consisting of (from the right) a 5’ terminal repeat (TR), CMV promotor, Cas9(H840A)-linker-M-MLV-RT (PE2), P2A, PuroR, bGH pA, hU6 promotor, pegRNA, and 3’ TR. **(B,C)** Integration of piggyPrime vectors containing the HEK3-CTTins, HEK3-Ains, HBB(E7V), FANCF-6Gdel, and PRNP-GtoT pegRNAs into HEK293T **(B)** and HeLa **(C)** cells resulting in increasing correct editing at target sites over time. Puromycin was applied at day 3 after transfection. **(D)** piggyPrime vectors can also successfully be integrated into the genome of K562 cells by transfection, resulting in increased correct editing over time. **(E)** pegRNA levels were determined at day 3 and 14 in K562 cells transfected with the HEK3-CTTins piggyPrime vector either with or without hyPBase. Only cells co-transfected with the piggyPrime vector and hyPBase-encoding plasmid DNA showed detectable pegRNA levels at day 14. **(F)** Editing rates were determined at multiple time points in K562 cells transfected with the HEK3-CTTins piggyPrime vector either with or without hyPBase. Only cells co-transfected with the piggyPrime vector and hyPBase-encoding plasmid DNA showed detectable correct editing. **(G)** The average copy-number of HEK3-CTTins piggyPrime vectors was determined in HEK293T and HeLa cells using ddPCR. **(H)** Schematic overview of piggyPrime vectors carrying both a nicking sgRNA (ngRNA) and a pegRNA (top) or dual pegRNAs (bottom). **(I)** Integration of HBB(E7V) piggyPrime vector carrying both a ngRNA and a pegRNA cassette resulted in markedly increased editing compared to HBB(E7V) piggyPrime vectors without a ngRNA. **(J)** Integration of piggyPrime vectors carrying both the HEK3-CTTins and HBB(E7V) pegRNA resulted in correct editing at both target sites, without compromising editing efficacy. Data and error bars show mean (*n* = 3) ± sd. Statistical significance was calculated using multiple unpaired *t*-tests with correction for multiple testing (*p < 0.03, **p < 0.002).

Next, we sought to develop a single-vector system that could deliver all the components of the prime editing system to cells in an easy and adaptable way. For this, we constructed a piggyBac vector containing PE2, a puromycin resistance gene, and a pegRNA Golden Gate cloning cassette for easy pegRNA cloning (Figure 2A; Supplementary Note S2). We then constructed piggyBac-PE2-pegRNA vectors (hereafter referred to as piggyPrime vectors) using five different pegRNAs (Supplementary Note S1), integrated them into the genome of both HEK293T and HeLa cells using hyPBase and established puromycin-resistant cell lines. At day 3 after transfection, all cell lines showed minimal prime editing activity with no detectable editing in the majority of piggyPrime-transfected cells (Figure 2B). However, at day 10 after transfection, editing could be observed in all cell lines, with editing rates ranging from 46 ± 1.5% to 98 ± 4% in HEK293T cells (Figure 1B) and from 32 ± 4% to 69 ± 0.6% in HeLa cells (Figure 2C). In accordance with our initial findings, editing rates kept increasing at all targets at day 17 and 24 after transfection, with some targets reaching 100% editing (Figure 2B). At day 24, the average editing rates across all 5 pegRNAs were 84.7 ± 15.5% and 63.6 ± 17.1% in HEK293T and HeLa cells, respectively. Furthermore, we successfully integrated the HEK3-CTTins and HBB(E7V) piggyPrime vectors into K562 cells by transfection and observed up to 46 ± 0.7% correct editing at day 24, despite the fact that editing could not be detected at day 3 after transfection (Figure 2D). For all targets across all cell lines, we did not detect any indel formation at day 24 after transfection (Supplementary Figure S2).

Additionally, we measured the level of pegRNA present in selected cell lines to confirm that the PE2-pegRNA cassette was stably integrated and expressed. We transfected K562 cells with piggyPrime vectors either with or without co-transfection of hyPBase-encoding plasmid DNA and found that only cells co-transfected with hyPBase had pegRNA levels that were detectable by ddPCR at day 14 (Figure 2E; Supplementary Figure S3A). Furthermore, only the cells that were co-transfected with hyPBase showed an increase in prime editing activity from day 3 to 14 (Figure 2F; Supplementary Figures S3B,C). We also determined the copy-number of the integrated HEK3-CTTins piggyPrime vectors and found that, on average, 6 and 5.4 copies were present in HEK293T and HeLa cells, respectively (Figure 2G). Hence, this confirmed that PE2 and pegRNAs were indeed stably expressed from integrated transposons, and that prolonged expression of PE2 and pegRNA was necessary to achieve increased levels of prime editing.

An increase in prime editing efficacy can be achieved by delivering a conventional sgRNA (termed a “nicking sgRNA” or “ngRNA”) in addition to the pegRNA, a method termed PE3 or PE3b (Anzalone et al., 2019). Therefore, we additionally constructed an HBB(E7V) piggyPrime vector capable of integrating a ngRNA cassette in addition to the PE2 and the HBB(E7V) pegRNA (Figure 2H). Notably, including a ngRNA within the HBB(E7V) piggyPrime vector led to markedly higher editing rates at the HBB target in HEK293T cells at day 3 and 10 after transfection (Figure 2I). We also constructed a piggyPrime vector encoding both HBB(E7V) and HEK3-CTTins pegRNAs (Figure 2H), which led to successful multiplexed editing of both the HBB and HEK3 target, without compromising editing efficacy at any of the targets (Figure 2J).

## Discussion

This study demonstrates, to the best of our knowledge, the first single-vector system capable of genomic integration of all the components of both the PE2 and PE3/PE3b system resulting in potent editing of up to 100% of targeted alleles. Editing efficacies were generally found to be lower in HeLa and K562 cells compared to HEK293T cells, which may partially reflect differences in plasmid transfection rates. Such differences between cell lines are in accordance with previous studies also reporting lower efficacy of prime editing in both HeLa and K562 cells (Anzalone et al., 2019; Nelson et al., 2021). Some concern could be raised in regard to generating cell lines with constitutively expressed genome editing effectors; however, the prime editing system has been shown to confer far less off-target editing than conventional CRISPR-Cas9, thus minimizing concerns of such undesired editing (Anzalone et al., 2019). Alternatively, potential unwanted off-target editing or by-product formation could be addressed by using controllable expression systems in the piggyPrime vector context. Additionally, in all experiments we were unable to detect any indel formation at day 24 after transfection by Sanger sequencing, even when including a ngRNA, which has been shown to increase indel formation of the PE system (Anzalone et al., 2019).

PiggyPrime represents a groundbreaking new approach for effective generation of transgenic model cell lines harboring disease-causing genetic variants or genes with desired genetic alterations. piggyPrime does not depend on homologous recombination and only requires a single pegRNA to be designed and cloned into a piggyPrime vector in a one-step Golden Gate cloning, thus aiding universal use. If desired, the piggyPrime system can furthermore accommodate a nicking sgRNA in addition to the pegRNA, which is expected to increase efficacy at targets of interest. Alternatively, the system can be multiplexed allowing two and potentially more pegRNAs to be integrated to generate complex disease models harboring multiple mutations. Our findings demonstrate potent prime editing leading to effective transgenesis in cells with prolonged expression of key prime editing components, suggesting that a longer time frame for prime editing is crucial for improved efficacy and common use. piggyPrime is easy adaptable to most proliferating cell types and is likely to become the standard technology for generation of cells with tailored genetic edits throughout the scientific community.

## Data Availability

The original contributions presented in the study are included in the article/Supplementary Material, further inquiries can be directed to the corresponding author.

## References

[B1] Anderson M. V. Haldrup J. Thomsen E. A. Wolff J. H. Mikkelsen J. G. (2021). pegIT - a Web-Based Design Tool for Prime Editing. Nucleic Acids Res. 49, W505–W509. 10.1093/nar/gkab427 34060619 PMC8265180

[B2] Anzalone A. V. Randolph P. B. Davis J. R. Sousa A. A. Koblan L. W. Levy J. M. (2019). Search-and-Replace Genome Editing without Double-Strand Breaks or Donor DNA. Nature 576 (7785), 149–157. 10.1038/s41586-019-1711-4 31634902 PMC6907074

[B3] Bloh K. Kanchana R. Bialk P. Banas K. Zhang Z. Yoo B.-C. (2021). Deconvolution of Complex DNA Repair (DECODR): Establishing a Novel Deconvolution Algorithm for Comprehensive Analysis of CRISPR-Edited Sanger Sequencing Data. CRISPR J. 4 (1), 120–131. 10.1089/crispr.2020.0022 33571043 PMC7898406

[B4] Chemello F. Chai A. Li H. Rodriguez-Caycedo C. Sanchez-Ortiz E. Atmanli A. (2021). Precise Correction of Duchenne Muscular Dystrophy Exon Deletion Mutations by Base and Prime Editing. Sci. Adv. 7 (18), eabg4910. 10.1126/sciadv.abg4910 33931459 PMC8087404

[B5] Holmgaard A. Askou A. L. Benckendorff J. N. E. Thomsen E. A. Cai Y. Bek T. (2017). *In Vivo* knockout of the Vegfa Gene by Lentiviral Delivery of CRISPR/Cas9 in Mouse Retinal Pigment Epithelium Cells. Mol. Therapy-Nucleic Acids 9, 89–99. 10.1016/j.omtn.2017.08.016 PMC5626917 29246327

[B6] Jang H. Jo D. H. Cho C. S. Shin J. H. Seo J. H. Yu G. (2021). Application of Prime Editing to the Correction of Mutations and Phenotypes in Adult Mice with Liver and Eye Diseases. Nat. Biomed. Eng. 10.1038/s41551-021-00788-9 34446856

[B7] Jinek M. Chylinski K. Fonfara I. Hauer M. Doudna J. A. Charpentier E. (2012). A Programmable Dual-RNA–Guided DNA Endonuclease in Adaptive Bacterial Immunity. Science 337 (6096), 816. 10.1126/science.1225829 22745249 PMC6286148

[B8] Jinek M. East A. Cheng A. Lin S. Ma E. Doudna J. (2013). RNA-Programmed Genome Editing in Human Cells. eLife 2, e00471. 10.7554/eLife.00471 23386978 PMC3557905

[B9] Kim Y. Hong S.-A. Yu J. Eom J. Jang K. Yoon S. (2021). Adenine Base Editing and Prime Editing of Chemically Derived Hepatic Progenitors Rescue Genetic Liver Disease. Cell Stem Cell 28, 1614–1624. 10.1016/j.stem.2021.04.010 33951479

[B10] Kumar M. Keller B. Makalou N. Sutton R. E. (2001). Systematic Determination of the Packaging Limit of Lentiviral Vectors. Hum. Gene Ther. 12 (15), 1893–1905. 10.1089/104303401753153947 11589831

[B11] Liu P. Liang S.-Q. Zheng C. Mintzer E. Zhao Y. G. Ponnienselvan K. (2021). Improved Prime Editors Enable Pathogenic Allele Correction and Cancer Modelling in Adult Mice. Nat. Commun. 12 (1), 2121. 10.1038/s41467-021-22295-w 33837189 PMC8035190

[B12] Liu Y. Li X. He S. Huang S. Li C. Chen Y. (2020). Efficient Generation of Mouse Models with the Prime Editing System. Cel Discov. 6 (1), 27. 10.1038/s41421-020-0165-z PMC7186222 32351707

[B13] Nelson J. W. Randolph P. B. Shen S. P. Everette K. A. Chen P. J. Anzalone A. V. (2021). Engineered pegRNAs Improve Prime Editing Efficiency. Nat. Biotechnol. 184 (22), 5635.5652.e29. 10.1038/s41587-021-01039-7 PMC8930418 34608327

[B14] Park S.-J. Jeong T. Y. Shin S. K. Yoon D. E. Lim S.-Y. Kim S. P. (2021). Targeted Mutagenesis in Mouse Cells and Embryos Using an Enhanced Prime Editor. Genome Biol. 22 (1), 170. 10.1186/s13059-021-02389-w 34082781 PMC8173820

[B15] Petri K. Zhang W. Ma J. Schmidts A. Lee H. Horng J. E. (2021). CRISPR Prime Editing with Ribonucleoprotein Complexes in Zebrafish and Primary Human Cells. Nat. Biotechnol., 1–5. 10.1038/s41587-021-00901-y 33927418 PMC8553808

[B16] Qian Y. Zhao D. Sui T. Chen M. Liu Z. Liu H. (2021). Efficient and Precise Generation of Tay–Sachs Disease Model in Rabbit by Prime Editing System. Cel Discov. 7 (1), 50. 10.1038/s41421-021-00276-z PMC8260710 34230459

[B17] Ran F. A. Hsu P. D. Wright J. Agarwala V. Scott D. A. Zhang F. (2013). Genome Engineering Using the CRISPR-Cas9 System. Nat. Protoc. 8 (11), 2281–2308. 10.1038/nprot.2013.143 24157548 PMC3969860

[B18] Schene I. F. Joore I. P. Oka R. Mokry M. van Vugt A. H. van Boxtel R. (2020). Prime Editing for Functional Repair in Patient-Derived Disease Models. Nat. Commun. 11 (1), 1–8. 10.1038/s41467-020-19136-7 33097693 PMC7584657

[B19] Shalem O. Sanjana N. E. Hartenian E. Shi X. Scott D. A. Mikkelsen T. S. (2014). Genome-scale CRISPR-Cas9 Knockout Screening in Human Cells. Science 343 (6166), 84–87. 10.1126/science.1247005 24336571 PMC4089965

[B20] Thomsen E. A. Rovsing A. B. Anderson M. V. Due H. Huang J. Luo Y. (2020). Identification of BLNK and BTK as Mediators of Rituximab‐Induced Programmed Cell Death by CRISPR Screens in GCB‐Subtype Diffuse Large B‐Cell Lymphoma. Mol. Oncol. 14 (9), 1978–1997. 10.1002/1878-0261.12753 32585766 PMC7463323

[B21] Yusa K. Zhou L. Li M. A. Bradley A. Craig N. L. (2011). A Hyperactive piggyBac Transposase for Mammalian Applications. Proc. Natl. Acad. Sci. 108 (4), 1531–1536. 10.1073/pnas.1008322108 21205896 PMC3029773

