# Supplementary material for: piggyPrime: High-Efficacy Prime Editing in Human Cells Using piggyBac-Based DNA Transposition
Source: Front Genome Ed. 2021 Nov 12;3:786893. doi: 10.3389/fgeed.2021.786893 (PMC8633390; doi:10.3389/fgeed.2021.786893)
Supplement: Supplementary file 1 [file DataSheet1.docx]

Supplementary Material

**Supplementary Figure 1**

Supplementary Figure 1 | piggyBac-mediated integration of PE2 and pegRNA cassettes using separate piggyBac vectors (indel rates). Accompanying indel rates in addition to correct editing rates for Figure 1D. Individual data points for each independent replicate are shown (n = 3).

**Supplementary Figure 2**

Supplementary Figure 2 | piggyBac-mediated integration of all prime editing components using a single-vector system (indel rates). Indel rates accompanying Figure 2B-D. Individual data points for each independent replicate are shown (n = 3).

**Supplementary Figure 3**

**
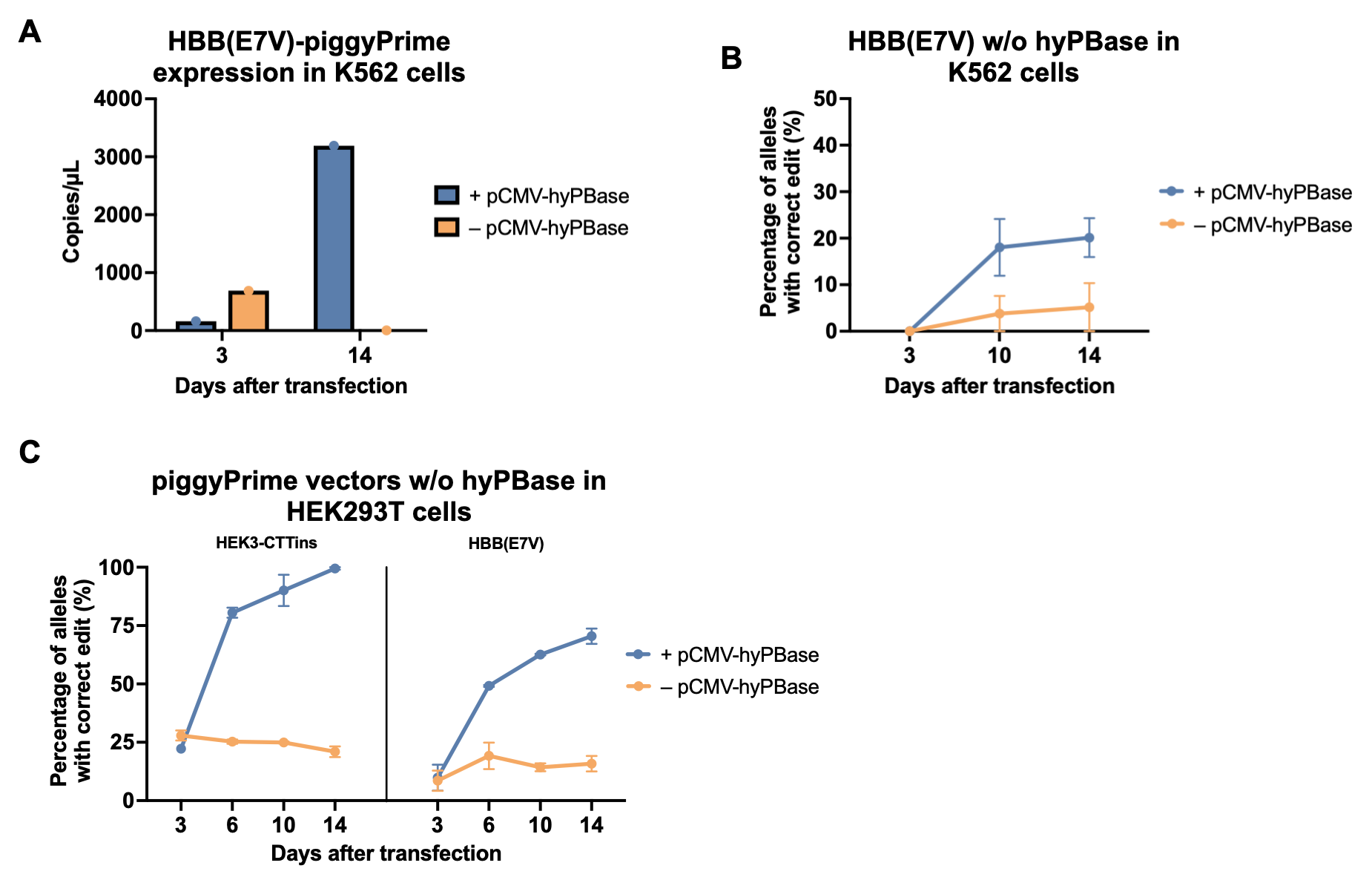
**

Supplementary Figure 3 | Integration versus transient expression of piggyPrime vectors in K562 and HEK293T cells. (A-B) K562 cells were transfected with the HBB(E7V)-piggyPrime vector both with and without co-transfection of pCMV-hyPBase. Only cells co-transfected with hyPBase showed detectable levels of pegRNA at day 14, confirming stable expression of the piggyPrime vector (A). This was also evident from the editing rates, which were higher in cells with integrated piggyPrime vectors (B). (C) HEK293T cells were transfected with indicated piggyPrime vectors either with or without pCMV-hyPBase, demonstrating that integration and stable expression of prime editing components were necessary to achieve high efficacy prime editing (n = 3).

**Supplementary Table 1: pegRNAs and ngRNAs used in this project (sequences)**

| Name | Spacer (5’-3’) | Extension (only for pegRNAs) (5’-3’) | PBS length (nt) | RTT length (nt) |
| --- | --- | --- | --- | --- |
| HEK3-CTTins | GGCCCAGACTGAGCACGTGA | TCTGCCATCAAAGCGTGCTCAGTCTG | 13 | 13 |
| HEK3-Ains | GGCCCAGACTGAGCACGTGA | TCTGCCATCATCGTGCTCAGTCT | 12 | 11 |
| FANCF-Gdel | GGAATCCCTTCTGCAGCACC | GGAAAAGCGATCAGGTGCTGCAGAAGGGATT | 15 | 16 |
| HBB(E7V) | GCATGGTGCACCTGACTCCTG | AGACTTCTCCACAGGAGTCAGGTGCAC | 13 | 14 |
| PRNP-GtoT | GCAGTGGTGGGGGGCCTTGG | ATGTAGACGCCAAGGCCCCCCACC | 12 | 12 |
| HBB(E7V)-ngRNA | GTAACGGCAGACTTCTCCAC | –––––––––––––––––––––––––––––––––––– | –––– | -––––– |

**Supplementary Table 2**: **Primers used for amplification and sequencing of genomic targets**

| Name | Sequence (5’-3’) |
| --- | --- |
| HEK3.FOR | ATGTGGGCTGCCTAGAAAGG |
| HEK3.REV | CCCAGCCAAACTTGTCAACC |
| HBB.FOR | GGGTTGGCCAATCTACTCCC |
| HBB.REV | GTCTTCTCTGTCTCCACATGCC |
| FANCF.FOR | AAGAACCTCTTTGTGTGGCGA |
| FANCF.REV | CTATAGCCATTGAAGCGCAGC |
| PRNP.FOR | CTGGGGGCAGCCGATAC |
| PRNP.REV | GACACCACCACTAAAAGGGC |

**Supplementary Table 3: Primers used for cloning of vectors used in this study**

| Plasmid | Fragment | Primer Name | Primer Sequence (5’-3’) |
| --- | --- | --- | --- |
| piggyPrime-ngRNA-pegRNA (multiplex) | hU6-ngRNA | pPmulti-pegRNA.FOR | GCGGTGGGCTCTATGGGCGGCCGCTCTAGAGAGGGCCTATTTCCCAT |
|  |  | pPmulti-ngRNA.FOR | TGGTGACCCGGGGGATCCACTAGTTCTAGACTTGCTATTTCTAGCTCTAA |
| piggyPrime-pegRNA1-pegRNA2 (multiplex) | hU6-pegRNA | pPmulti-pegRNA.FOR | GCGGTGGGCTCTATGGGCGGCCGCTCTAGAGAGGGCCTATTTCCCAT |
|  |  | pPmulti-pegRNA.REV | TGGTGACCCGGGGGATCCACTAGTTCTAGACTTTTATCGAATTCCTGCAGC |
| pPBT-PE2 | CMV-PE2 | PE2.FOR | TTTTCTTGTTATAGATATCAAGCTTGACATTGATTATTGACTAGTTATTAATAG |
|  |  | PE2.REV | GATCCCCCGGGGGATCTACATTAGACTTTCCTCTTCTTCTTG |
| pPBT-PE2-PGK-Blast | PGK-Blast | PGK-Blast.FOR | GGGCTCTATGGGCGGCCGCTGGGGTTGGGGTTGCGCCT |
|  |  | PGK-Blast.REV | GAAGGCACAGTTAGCCCTCCCACACATAACCAGAGG |
|  | bGH-pA | bGH-pA.FOR | GGAGGGCTAACTGTGCCTTCTAGTTGCCAG |
|  |  | bGH-pA.REV | TGCAGCCCGGGGCACTAGTTCTAGACCATAGAGCCCACCGCATC |
| pPBT-pegRNA_GG-Puro | pegRNA-GG-acceptor (BsmBI) | pegRNA-GG.FOR | TATATATATATTTTCTTGTTATAGATATCAAGCTTCCAGAGAGGGCCTATTTCCCA |
|  |  | pegRNA-GG.REV | CTTTGCACCCGGGGATCTAGTTACGCCAAGCTTAAAAAAACGAG |
|  | EF1a-Puro | Puro.FOR | CGTAACTAGATCCCCGGGTGCAAAGATGGATAAAG |
|  |  | Puro.REV | AACTTTTATCGAATTCCTGCAGCCCGGGTTAACGCGTTCAGGCACCGG |
| pPBT-PE2-PuroTK-pegRNA_GG | Linker-M-MLV-RT | MLV-RT.FOR | CTCTGGAGGATCTAGCGGAG |
|  |  | MLV-RT.REV | CGCCGGATCCGACTTTCCTCTTCTTCTTGGGC |
|  | P2A-PuroTK-pA | PuroTK.FOR | GAGGAAAGTCGGATCCGGCGCAACAAACTT |
|  |  | PuroTK.REV | ATAATGGTGACCCGGGGGATCCACTAGTTCTAGAGCGGCC |
|  | pegRNA-GG-acceptor (BsmBI) | pegRNA-GG_2.FOR | ATCCCCCGGGTCACCATTATCGTTTCAGACCCAC |
|  |  | pegRNA-GG_2.REV | TTATCGAATTCCTGCAGCCCAAAAAAACGAGACGTCCCTATCAG |

**Supplementary Table 4: Primers and probes used for ddPCR**

| Name | Sequence (5’-3’) |
| --- | --- |
| PuroR_fwd | CAAGAACTCTTCCTCACGCG |
| PuroR_rev | GCCGATCTCGGCGAACA |
| PuroR_probe | FAM-ACATCGGCAAGGTGTGGGTCG-BHQ |
| ALB_fwd | GCTGTCATCTCTTGTGGGCTGT |
| ALB_rev | ACTCATGGGAGCTGCTGGTTC |
| ALB_probe | HEX-CCTGTCATGCCCACACAAATCTCTCC-BHQ |
| CTT-pegRNA_fwd | GCCCAGACTGAGCACGTGAG |
| HBB-pegRNA_fwd | CATGGTGCACCTGACTCCTGG |
| pegRNA_rev | CCGACTCGGTGCCACTTT |
| pegRNA_probe | FAM-AAATAAGGCTAGTCCGTTATCAACTT-BHQ |

**Supplementary Note 1:** **pegRNAs and ngRNAs used in this project (descriptions)**

The 5 different pegRNAs used in this study are described in the following and the corresponding sequences are listed in Supplementary Table 1. **HEK3-CTTins**: Designed to insert a CTT trinucleotide at the *HEK3* site at position +1 relative to the PE2-induced cut-site (Anzalone et al., 2019). **HEK3-Ains:** Designed to insert a single A nucleotide at the *HEK3* site at position +1 relative to the cut-site (Anzalone et al., 2019). **FANCF-Gdel:** Designed to delete a G in the gene encoding the Fanconi anemia group F protein (*FANCF*) at position +6 relative to the cut-sitse (Anzalone et al., 2019). **HBB(E7V):** Designed to induce an A-to-T substitution in the *HBB* gene. This results in glutamine 7 being converted to valine (E7V), which is the most common cause of sickle-cell disease (Anzalone et al., 2019). The ngRNA used for the multiplexed piggyPrime vector was designed to induce a nick on the opposite strand at position +5 relative to the pegRNA-induced cut-site. The ngRNA was designed using pegIT (Anderson et al., 2021) according to the PE3b system (Anzalone et al., 2019). **PRNP-GtoT:** Designed to install a G to T transversion into the *PRNP* gene, giving rise to a PRNP(G127V) mutation that confers resistance to prion disease in humans (Anzalone et al., 2019).

| pegRNA | Targeted gene/locus | Intended edit | Location* |
| --- | --- | --- | --- |
| HEK3-CTTins | *HEK3* (Chromosome 9) | CTT insertion | 1 |
| HEK3-Ains | *HEK3* (Chromosome 9) | A insertion | 1 |
| FANCF-Gdel | *FANCF* | G deletion | 6 |
| HBB(E7V) | *HBB* | A to T substitution | 4 |
| PRNP-GtoT | *PRNP* | G to T substitution | 6 |

* Location of intended edit numbered by base pairs downstream from the PE2-induced nick site. 1 indicates the first bp directly downstream of the nick site.

**Supplementary Note 2: Cloning of pegRNAs into piggyPrime vectors**

Cloning of pegRNAs into piggyPrime vectors is easy and efficient and can be accomplished using a slightly modified version of the cloning protocol published by Anzalone and coworkers (Anzalone et al., 2019). For cloning of piggyPrime vectors, BsmBI should be used, and no pre-digestion of the vector is necessary. We recommend using 500 ng plasmid per 10 μL reaction, to account for the larger size of the piggyPrime vectors. Furthermore, we recommend extending the number of Golden Gate cycles from 8 to 30 to increase the efficiency of piggyPrime pegRNA clonings. The cloning method is directly compatible with cloning of pegRNAs into pPBT-pegRNA_GG-Puro, if the dual-vector system is to be used.

**Supplementary References**

Anderson, M.V., Haldrup, J., Thomsen, E.A., Wolff, J.H., and Mikkelsen, J.G. (2021). pegIT-a web-based design tool for prime editing. *Nucleic Acids Research*.

Anzalone, A.V., Randolph, P.B., Davis, J.R., Sousa, A.A., Koblan, L.W., Levy, J.M., et al. (2019). Search-and-replace genome editing without double-strand breaks or donor DNA. *Nature* 576(7785)**,** 149-157. doi: 10.1038/s41586-019-1711-4.
